# Visualization of Chronic Myocardial Infarction Using the Intravascular Contrast Agent MS-325 (Gadofosveset) in Patients

**DOI:** 10.1100/2012/236401

**Published:** 2012-03-12

**Authors:** Thomas Thouet, Bernhard Schnackenburg, Thomas Kokocinski, Eckart Fleck, Eike Nagel, Sebastian Kelle

**Affiliations:** ^1^Department of Internal Medicine/Cardiology, German Heart Institute Berlin, 13353 Berlin, Germany; ^2^Philips Healthcare, 20099 Hamburg, Germany; ^3^Division of Imaging Sciences and Medical Engineering, Department of Cardiovascular Imaging, King's College London, London 5E1 7EH, UK

## Abstract

*Aims*. The aim of this study was to evaluate the potential of visualizing chronic myocardial infarction in patients using the intravascular CA MS-325 (gadofosveset, EPIX Pharmaceuticals, Mass, USA). *Methods*. Nine patients were enrolled in a clinical phase II multicenter trial for MRCA and perfusion imaging using MS-325. They had objective evidence of chronic myocardial infarction as visualized by previously performed late gadolinium (Gd) enhancement imaging (LGE) with a conventional extracellular Gd-DTPA CA (Magnevist, Bayer Healthcare, Germany, 0.2 mmol/kg/body weight) serving as reference standard. A prepulse-optimized LGE study was performed immediately and at several time points after injection of MS-325 (0.05 mmol/kg/body weight). The number and localization of segments demonstrating LGE with MS-325 as well as signal intensities were compared with the reference standard (Gd-DTPA). *Results*. Using MS-325, LGE could be detected at every time point in all 9 patients. The accuracy of LGE with MS-325 as compared to LGE with Gd-DTPA was highest 54 ± 4 minutes after contrast injection, resulting in a sensitivity of 84% with a specificity of 98%. *Conclusion*. The intravascular CA MS-325 has the potential to visualize chronic myocardial infarction. However, in comparison with Gd-DTPA, the transmural extent and the number of segments are smaller.

## 1. Introduction

Intravascular blood pool contrast agents (CAs) have been designed and studied in order to improve MR coronary artery imaging (MRCA) and peripheral MR angiography [1–8]. A possible approach is the use of gadolinium-based albumin-bound CA that has longer residence time in the blood pool, while the free, unbound components behave in a similar manner to extracellular media. The potential of such new aspects for the detection of myocardial scar has been studied in various animal models [9–11] with promising results. So far, no data for scar detection using intravascular CA in humans have been described.

Infarct size and its transmural extent are important predictors for the likelihood of functional recovery and patient prognosis [12–14]. Magnetic resonance late gadolinium enhancement (LGE) using a gadolinium-based (Gd) extravascular contrast agent is a reliable technique for the detection of myocardial scar and can be used to distinguish between reversible and irreversible myocardial damage [15–17]. The technique can be regarded as reference standard in clinical routine, and its reproducibility has been demonstrated [18].

We evaluated the potential of the intravascular CA MS-325 for detection of myocardial scar in patients with chronic myocardial infarction.

## 2. Materials and Methods

### 2.1. Study Protocol

The results presented here were obtained as a single-center protocol within a multicenter phase II trial for feasibility and sequence optimization of MS-325 (gadofosveset; Vasovist) for combined MR myocardial perfusion imaging and MRCA. The study protocol was approved by the Charité and Virchow-Klinikum Institutional Ethics Committee, and all subjects gave informed consent prior to the cardiovascular magnetic resonance (CMR) examination. Patients were then scheduled for MRCA and perfusion imaging using MS-325. LGE measurements were performed in addition to the multicenter protocol.

### 2.2. Patient Population

A total of nine patients (8 males, mean age 56 years, range 35–66 years) were included. Patients were enrolled if they had evidence for chronic myocardial infarction as determined by a previous clinically indicated CMR examination including late gadolinium enhancement imaging using the extracellular CA (Gd-DTPA, Magnevist, Bayer Healthcare, Germany, 0.2 mmol/kg/body weight) at 1.5 Tesla MR (Philips, Intera, Best, The Netherlands). The mean infarct age was 36 months (3–134 months), and the X-ray coronary angiography performed within 3 months before or one month after MR exam showed reperfusion of the supplying coronary artery of the infarct area in 6 patients, chronic occlusion in 2, and partial reperfusion and occlusion in 1 patient. Patients were not included if they had a history of coronary artery bypass graft operation or presented with a severe reduction of left ventricular function (LV-EF < 30%) or severe valvular heart disease, were clinically unstable (e.g., unstable angina pectoris), had any medical history of anaphylactoid reaction, or had any contraindication for MRI examination.

### 2.3. MS-325

MS-325 is an intravascular contrast agent that has been designed and verified for MR angiography of the cardiovascular system [1–5] and has been approved for MR angiography of abdominal and peripheral vessels. It is characterized by a low molecular weight of 975.88 kD and low viscosity of 3.0 cP. It is constituted of a diphenylcyclohexyl group attached to a gadolinium (Gd) chelate by a phosphodiester linkage. Its reversible binding to human serum albumin (85–95%) has two major effects: first, vascular retention and low volume of distribution leading to a half-life of 16 hours; second, the binding to albumin reduces the molecular tumbling rate and induces better electron-nuclear interaction between the Gd and water protons resulting in a high relaxivity of 30–50 mmol^−1 ^sec^−1^ (at 37°C; depending on *B_0_*). Thus, compared to Gd-DTPA, 5 to 10 times higher relaxivities can be observed depending on magnetic fields and concentrations [1–5]. The unbound MS-325 behaves similarly to extracellular CA with diffusion into the extracellular compartment. Despite the missing interaction with albumin, unbound MS-325 has a relaxivity of 6.5 mmol^−1 ^sec^−1^ [19] compared to other extracellular CA (e.g., Gd-DTPA 3.5 mmol^−1 ^sec^−1^).

### 2.4. MR Imaging Protocol

All examinations were performed with a 1.5 Tesla MR Scanner (Intera, Philips Medical Systems, Best, The Netherlands) using a dedicated phased-array cardiac coil (Sense-cardiac coil, Philips, Best, The Netherlands). Patients were placed in a relaxed supine position and after a rapid survey to determine the exact heart axis, cine loops in 3 short-axis (SA) planes (apical, equatorial, and basal), and 3 long-axis (LA) planes (4-, 3-, and 2-chamber view) were acquired for further planning.

Reference LGE imaging, where LGE using an extracellular CA (Gd-DTPA; Magnevist, Bayer Healthcare, Germany, 0.2 mmol/kg/body weight) was defined as the reference, was performed using a prepared turbo gradient echo imaging sequence (3D, TR shortest; TE shortest; flip angle 15°) with a spatial resolution of 1.4 × 1.1 × 5 mm in end-expiratory breath-hold position. Prior to each LGE measurement, a look-locker measurement [20] was performed. Minimum time between scanning a patient with Gd-DTPA and MS-325 was 24 hours.

The weight adapted bolus of MS-325 of 0.05 mmol/kg/body weight was administered through a power injector (MedRad Inc., Indianola, US) with a flow rate of 1.5 mL/s immediately followed by a saline flush of 30 mL (1.5 mL/s). LGE measurements with MS-325 were performed in SA and LA using an inversion-prepared turbo gradient echo imaging sequence (3D, TR 4 ms; TE 1.95 ms; flip angle 15°) with a spatial resolution of 1.29 × 1.29 × 5 mm in end-expiratory breath-hold position. Prior to each LGE measurement with MS-325, a look-locker measurement [20] was performed to determine the individually adapted inversion prepulse delay for optimal suppression of myocardial signal. LGE imaging was performed at 3 time points (*t*_1_, *t*_2_,  *t*_3_). *t*_1_ was 4 ± 1 minutes after CA injection. *t*_2_ was performed after contrast-enhanced MRCA. Since the duration of MRCA depends on heart rate and the efficiency of breathing motion suppression (navigator efficiency) *t*_2_ varied between 17 and 37 minutes with an average of 26 ± 7 minutes after CA injection, *t*_3_ was set to 54 ± 4 minutes after injection.

### 2.5. MR Image Analysis

All MR images were analyzed on a dedicated workstation (ViewForum, Release 4.1; Philips, Best The Netherlands) and evaluated by two experienced MR readers. The presence of myocardial scar was visually assessed in a 16-segment model in 3 short axis slices (6 segments: basal, 6 segments: medial, and 4 segments: apical). Transmurality of infarcted segments was assessed in the short-axis slices using a 4-point scale (1: 1–25% transmural enhancement; 2: 26–50%; 3: 51–75%; 4: 76–100%).

At every time point, signal intensities were determined for regions of interest placed inside the enhanced area, in the left ventricular cavity (corresponding to blood pool), and in healthy myocardium. The following values were determined: mean, minimum, maximum, and standard deviation of the signal intensity.

### 2.6. Statistical Methods

Transmurality of the enhanced segments with MS-325 was normalized to the degree of transmurality previously detected with the extracellular CA and compared using a Wilcoxon signed-rank test. The number of detected enhanced segments for both methods is given in absolute values and percentage of the total number of assessable segments. Signal intensities were compared using the mean values, and the contrast between enhanced area, myocardium, and blood pool was calculated as follows: *C*_1,2_ = (SI_1_ − SI_2_)/(0.5 × (SI_1_ + SI_2_) [21]. Mean values, standard deviations, and *P* values were calculated for each dataset. The results were assumed to be statistically significant with a *P* value <0.05.

## 3. Results

### 3.1. Number of Enhanced Segments

**Figure 1:**
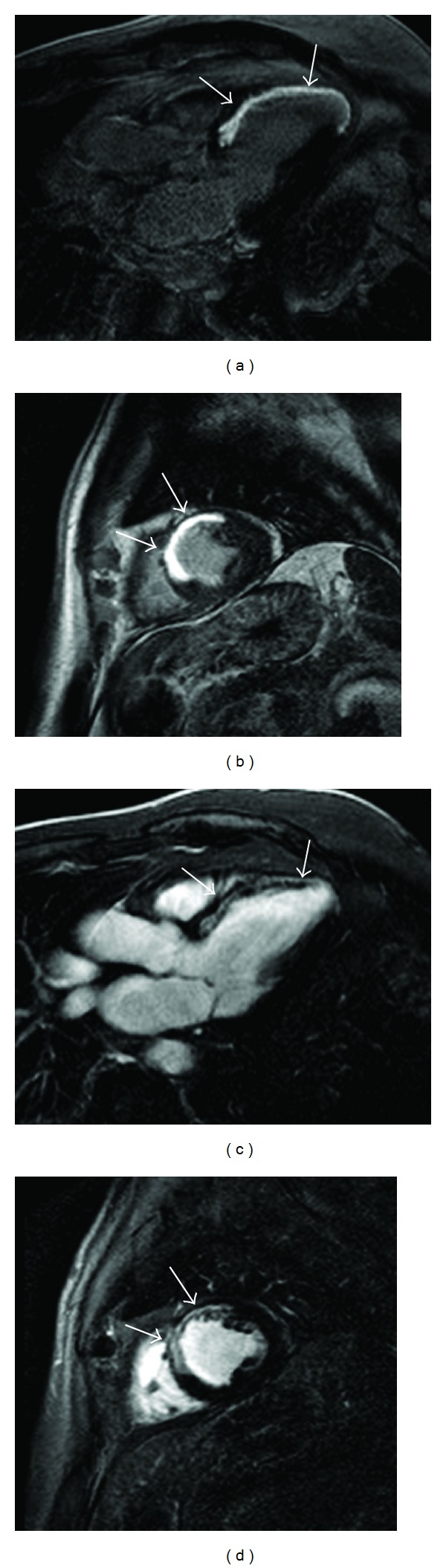
Transmural LGE detected with the extracellular CAs in long-axis and short-axis view (a and b) compared with MS-325 LGE (c and d). Both CA show a clear contrast between the enhanced area and the surrounding normal myocardium. However, blood pool signal is brighter for MS-325 related to higher relaxivity and vascular retention.

All segments (9 × 16 = 144) could be evaluated with Gd-DTPA and MS-325 after injection (Figure 1). With the extracellular CA 55, segments were classified as having LGE. Using MS-325, LGE could be detected at every time point in all 9 patients.

At *t*_1_, 45 segments (per segment sensitivity 82%) showed LGE in identical segments as Gd-DTPA, 10 segments were false negative, and 1 segment was false positive with MS-325 (per segment specificity 99%). At *t*_2_, 41 segments were true positive (per segment sensitivity 75%) with no false positive and 14 false negative (per segment specificity 100%). At *t*_3_, 46 segments were true positive (per segment sensitivity 84%), 9 false negative and 1 false positive (per segment specificity 98%).

### 3.2. Transmurality

**Figure 2:**
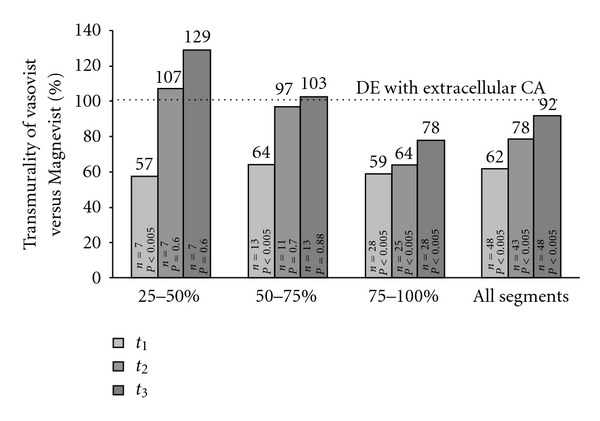
This figure shows the mean transmurality detected with MS-325 as a percentage of the transmurality previously detected with the extracellular CA (LGE). The columns show the results grouped for different degrees of transmurality with extracellular CA. Transmurality at *t*_1_ was underestimated with MS-325 independent of transmurality, while transmurality of 25–50% at *t*_2_ (107%) and transmurality of 25–50% and 50–75% at *t*_3_ were overestimated (129% and 103%, resp.) using MS-325.

The reference LGE imaging showed a mean transmurality of 3.2 ± 0.8. Thirteen segments (9%) showed 25–50% transmural enhancement, 14 segments (9%) 50–75%, and 32 segments (21%) 75–100%. None of the segments was classified as having LGE of 1–25% transmurality. Using MS-325, the transmurality was significantly underestimated at all time points with a mean transmural extent of 2.0 ± 0.75 (*P* < 0.0005, 62% of LGE with extracellular CA) at *t*_1_, 2.6 ± 0.79 at *t*_2_ (*P* < 0.0005, 78%), and 3 ± 0.93 at *t*_3_ (mean 3.0, 92% *P* < 0.0005). Interestingly, using MS-325, a tendency towards overestimation was found at *t*_2_ and *t*_3_ for segments with 25–50% transmural extent identified by Gd-DTPA, as well as at *t*_3_ for segments with 50–75% transmural extent (Figure 2).

### 3.3. Time-Intensity Curves

**Figure 3:**
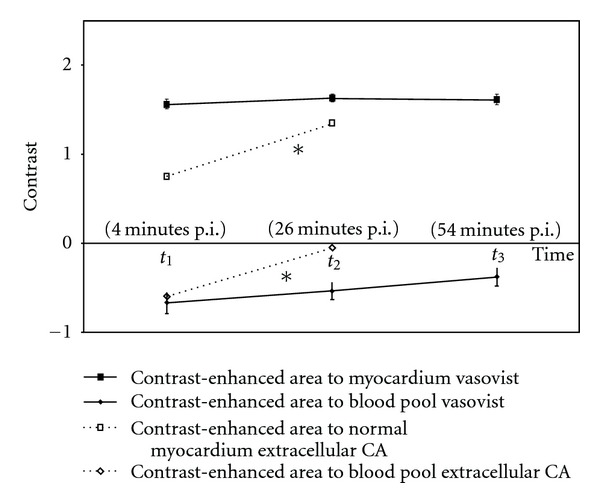
Contrast between enhanced area and normal myocardium increased from *t*_1_ to *t*_3_ as enhanced segments showed increasing signal intensities and normal myocardium is assumed to be effectively nullified using the correct inversion prepulse (look locker approach). As blood pool consistently showed the highest signal intensity over the time, blood pool-enhanced area contrast decreased from *t*_1_ to *t*_2_ and *t*_3_ due to increasing SI in the enhanced area and nearly constant SI of the blood pool. The dotted line (∗) shows data for the extracellular CA Gd-DTPA extracted from a previous study [22]. Using Gd-DTPA (0.2 mmol/kg body weight), the contrast between enhanced area and normal myocardium increased continuously 4 minutes (*t*_1_) and 26 minutes (*t*_2_) after CA administration. Contrast between enhanced area and blood pool decreased from *t*_1_ to *t*_2_ (prepulse delay 275 ms, TE 3.6 ms, TR 8 ms, flip angle 15°).

Averaged signal intensities at each time point for MS-325 are given in Table 1. The contrast between enhanced area and myocardium increased to 1.56 ± 0.06  (1.35–1.77) at *t*_1_, 1.62 ± 0.04  (1.4–1.76) at *t*_2_, and 1.61 ± 0.06  (1.3–1.8) at *t*_3_. The contrast between enhanced area and blood pool decreased to −0.7 ± 0.11  (−0.97–0.03) at *t*_1_, −0.54 ± 0.1  (−0.92–0) at *t*_2_, and −0.38 ± 0.1  (0.72–0) at *t*_3_ (Figure 3).

## 4. Discussion

Our study shows considerable differences between LGE performed with a standard extracellular contrast agent (Gd-DTPA) versus a novel intravascular contrast agent (MS-325, gadofosveset). The accuracy of LGE with MS-325 as compared to LGE with Gd-DTPA was highest 54 ± 4 minutes after contrast injection resulting in a sensitivity of 84% with a specificity of 98%. Transmurality of LGE was significantly underestimated with the intravascular agent.

### 4.1. Late Gadolinium Enhancement

The present study shows that MS-325 enhances myocardial scar somewhat similarly to extracellular contrast agent. However, the segmental analysis showed considerable disagreement between intra- and extravascular contrast media. With some exceptions for subendocardial infarcts (<50% transmural enhancement), there was significant underestimation of the number of infarct segments and the transmurality of the segments compared to extracellular CA.

Signal intensities of the blood pool remained nearly constant with a small initial increase after 26 minutes followed by a slight decrease after 54 minutes, while SI of the enhanced area continuously increased (Table 1). These data agree with the characteristics of MS-325 published by Parmelee et al. [1] and the results from other albumin-bound intravascular CA [11] and can be explained by two principal mechanisms: the high level of plasma albumin binding leads to vascular retention and consistent signal from blood pool. On the other hand, MS-325 binds reversibly to albumin and 5–15% is unbound. This portion of CA behaves similarly to extracellular CA and diffuses into the extracellular compartment, leading to increasing contrast between normal myocardium and progressively enhancing scar tissue.

The relationship among infarct age, size, CA dosage, distribution, and timing has been established for extracellular CA [23–25], but for intravascular CA, little information has been provided as yet. Recently, Saeed et al. [26] performed LGE imaging in 8 pigs at 10, 20, and 40 after administration of 0.026 mmol/kg P792 (Vistarem), an intravascular, gadolinium-based CA with a plasma half-life ranging from 22 minutes in mice to 29 minutes in rats and 41 minutes in rabbits [27]. The chemical structure of P792 with a Gd-based compound (DOTA) provided with hydrophilic arms combines a high relaxivity with vascular retention and restriction from fast diffusion out of normal microvessels.

Our data are comparable to those found in previous studies using other intravascular CA that showed good agreement between extracellular CA, intravascular CA, and histomorphology [10]. Flacke et al. report good delineation of nonreperfused myocardial infarction with a long imaging window due to long blood pool persistence but relatively low CNR and slight overestimation compared to extracellular CA in a rat model 1 week after infarction [10].

The transmural extent and signal behavior using MS-325 may be influenced by the following factors.

In contrary to P792, vascular retention of MS-325 is related to the binding to plasma albumin, and diffusion of the unbound component to the extracellular compartment can be observed where MS-325 behaves similarly to extracellular CA. In contrary to Flacke et al. [24], our data rather suggest an underestimation of the transmurality of infarcted segments, particularly early after CA injection. This may be attributed to the fact that the mean infarct age in our study group was 36 months. The histological correlative for the detected enhancement may thus considerably vary compared to that of Flacke et al. [24]. However, Flacke et al. are the first authors to describe the detection of myocardial delayed enhancement using MS-325, compared to a conventional extravascular gadolinium-based CA. To which extent the infarct age and subsequent histomorphological changes influence the dimensions of LGE using MS-325 cannot be derived from our data.

The unbound portion of MS-325 diffuses into the extracellular compartment of myocardial scar where its relaxivity is supposed to decrease due to the missing interaction with albumin [28]. However, Caravan et al. showed that *in vitro* unbound MS-325 has still a higher relaxivity of 6.6 mmol^−1 ^sec^−1^ compared to Gd-DTPA (3.5 mmol^−1 ^sec^−1^) [19]. Additionally, at the clinical dose of 0.05 mmol/kg used in this study, saturation of albumin may already occur and will lead to a larger distribution volume including the scar tissue [29]. Consequently, the exact amount of MS-325 passing the capillary membrane, the washout kinetics, and its signal behavior are not known.

## 5. Limitations

In this study protocol, parallel imaging (Sense technique) was used as it allows higher spatial resolutions and shorter scan times, suitable for dynamic contrast-enhanced perfusion imaging. Thus, the computation of signal-to-noise ratios, expedient for tracing of the signal in blood pool, scar, and myocardium after CA administration, was not applicable, as SNR may vary within the field of view and between repeat measurements at different time points. Nonetheless, the tracing of contrast as performed in this study gives an approximate estimation of MS-325 influence on signal intensities.

This small study with a limited sample size is a single-center spin-off of a clinical phase II multicenter trial for MRI-based feasibility and sequence optimization of myocardial perfusion and coronary angiography study. The clinical application could be in a protocol designed for imaging of coronary arteries or veins using MS-325.

## 6. Conclusions

Myocardial scar can be detected with the intravascular contrast agent MS-325. All patients with scar revealed by Gd-DTPA contrast-enhanced MR imaging showed myocardial LGE with MS-325. However, both the number of segments and the transmurality of scar were underestimated with the intravascular contrast agent in comparison to the extracellular CA.

## Figures and Tables

**Table 1:** Averaged signal intensities (SIs) in the enhanced area, blood pool, and normal myocardium at each time point. Values are expressed as mean ± standard deviation.

	Time point		
SI	*t*_1_	*t*_2_	*t*_3_
Enhanced area	911 ± 136	1073 ± 112	1254 ± 139
Blood pool	1767 ± 86	1818 ± 73	1787 ± 46
Normal myocardium	122 ± 19	113 ± 9	124 ± 12
